# Revisiting CD28 Superagonist TGN1412 as Potential Therapeutic for Pediatric B Cell Leukemia: A Review

**DOI:** 10.3390/diseases6020041

**Published:** 2018-05-19

**Authors:** Katelyn E. Brown

**Affiliations:** Department of Biomedical Sciences, Colorado State University, Fort Collins, CO 80523, USA; kbrown10@rams.colostate.edu; Tel.: +1-303-204-4476

**Keywords:** clinical trials, pediatric therapeutics, B cell leukemia

## Abstract

Pediatric acute lymphoblastic leukemia (ALL) represents the most common pediatric cancer diagnosis, with numbers rising gradually every year. This paper proposes a novel therapeutic agent for pediatric ALL on the basis of a failed clinical drug trial in 2006. TGN1412 was a promising therapeutic agent that yielded outstanding results in both in vitro studies and animal trials. It is a CD28 superagonist monoclonal antibody that activates T regulatory (T_Reg_) cells in the absence of costimulation of the T cell receptor (TCR) by an antigen-presenting cell. This drug was intended as a solution to T cell deficient diseases such as B cell leukemia and autoimmune diseases such as rheumatoid arthritis. When phase I clinical trials were conducted, all volunteers that received the drug experienced severe cytokine release syndrome (CRS) and faced multiple-organ failure within hours. TGN1412 was reassessed and re-entered clinical trials as a therapeutic for rheumatoid arthritis. A new assay was developed to better quantify T cell response, and volunteers in this trial experienced no pro-inflammatory cytokine release. This essay analyzes how misinformation contributed to the failure of TGN1412 in clinical trials and how revisiting this therapeutic could yield a novel treatment for pediatric B cell leukemia.

## 1. Introduction

Pediatric B cell leukemia is a serious issue, with case numbers rising annually [1]. Approximately 2670 children aged 0–14 were diagnosed in 2014, with that number dropping dramatically to 410 for ages 15–19 [2]. The vast majority of diagnoses occur in children younger than 20 [2].

TGN1412 is a CD28 superagonist monoclonal antibody that preferentially activates regulatory T (T_Reg_) cells [3]. CD28 is a costimulatory molecule on the surface of naive T cells, which binds to B7 on antigen-presenting cells for activation. Generally, for the activation of a T cell, an antigen-presenting cell (such as a dendritic cell) must also stimulate the highly specific T cell receptor (TCR) by presenting its specific antigen on its class II major histocompatibility (MHC)complex. The presence of this costimulatory process helps to ensure that T cells are not incorrectly activated at any point. This is a key regulatory component in preventing self-recognizing T cells from accidentally becoming activated. TGN1412 acts by binding to CD28 on T_Reg_ cells, causing them to activate and proliferate in the absence of costimulation of the TCR [3].

This capability piqued researchers’ interests in using this superagonist antibody as a therapeutic agent, as T_Reg_ cells are highly important in preventing autoimmune diseases [3]. This capability indicated that TGN1412 could be instrumental in helping to prevent autoimmune diseases such as rheumatoid arthritis. Additionally, the therapeutic could theoretically be used in diseases with chronically low numbers of T cells, such as B cell leukemia [4].

TGN1412 showed promising potential in both in vitro studies and animal trials. Cynomolgus monkeys were used, as their CD28 molecules have 100% homology with that of the human TCR in both intracellular and extracellular domains. In vitro studies showed that TGN1412 bound to CD28 molecules on human and monkey T_Reg_ cells with incredibly similar affinities [5]. Animal trials showed that TGN1412 induced significant T_Reg_ cell response that was well tolerated in the cynomolgus bodies; concentrations of interleukins 2, 5, and 6 also all transiently increased. No drug-related adverse effects were observed in the animal models [5].

The phase I clinical trial was a randomized, double-blind procedure with eight healthy male volunteers of varying ages [6]. The drug was administered intravenously to six of the volunteers, and two were given a placebo. The full dosage was given at the time of injection, about 10 times more quickly than during the animal trials. The dosage was about 500 times more dilute than that of the animal studies [6].

Each volunteer was administered the drug at 10 min intervals from one another. Within the first 20 min, the first volunteer complained of a headache; the medical team kept him under close supervision but continued to administer the drug to the other volunteers. Symptoms continued to develop in the first volunteer, and the other volunteers soon began reacting in a similar manner; the most notable symptoms included excruciating back pain, migraines, vomiting, hypotension, and extreme fever. These symptoms developed in all volunteers within the first hour of receiving the drug [6].

The health of the volunteers continued to decline over the next 5 h. Because this was a novel therapeutic, the medical team did not administer additional drugs to try to relieve the symptoms. They were unsure of how long the symptoms would persist and how severe they would continue to be, and potential adverse drug interactions were unclear [6].

When it became clear that the volunteers were not improving and continued to deteriorate quickly, they were moved to a nearby Intensive Care Unit (ICU) in Northwick Park Hospital. The medical staff there determined that all volunteers were facing multiple-organ failure, but the exact cause was still unknown. Physicians noted that from the outside, the volunteers exhibited many symptoms associated with sepsis, which indicated that the injected TGN1412 may have been contaminated. Another physician discovered that a low-risk side effect of TGN1412 was severe cytokine release syndrome (CRS), also referred to as “cytokine storm” [6].

The physicians were faced with a dilemma here; if the volunteers were suffering from cytokine storm, immunosuppressants would likely improve their state dramatically. However, if the volunteers were battling sepsis, perhaps due to contamination of the drug, immunosuppressants would cause their health to decline even further. Physicians chose to treat the volunteers as if they were experiencing cytokine storm and administered immunosuppressants [6].

Within 24 h, the health of all the volunteers significantly improved. Four were released from the ICU within a week, and the fifth was released after 3 weeks. One volunteer remained in the hospital for the next 4 months [6]. He experienced disseminated intravascular coagulation, resulting in the loss of all toes and most fingers [7].

CRS occurs when a massive amount of unregulated pro-inflammatory cytokines are released into the body [8]. In this case, it is closely linked to multiple-organ dysfunction syndrome (MODS). In the first phase of CRS, pro-inflammatory cytokines are released that stimulate activation and differentiation of lymphocytes. Additionally, these cytokines promote vasodilation and increase the expression of adhesion molecules on the surface of endothelial cells. Neutrophils are also activated and recruited in this process [9]. When neutrophils are activated, the second phase of CRS begins; both neutrophils and cytotoxic T lymphocytes (CTLs) are recruited, and their actions result in endothelial damage. This creates a positive feedback loop; as more inflammation is caused by these cells, more cytokines are released to facilitate the activation of additional leukocytes. Endothelial damage increases and, when paired with a gross release of unregulated pro-inflammatory cytokines, results in multiple-organ failure. It is theorized that TGN1412 initiated this response by preferentially binding and over-activating T_Reg_ cells [6].

## 2. Discussion

For many years, the causation of the cytokine storm remained largely unknown; there was speculation of contamination or other incidences of foul play, but these were quickly disproven by the Medicines and Healthcare products Regulatory Agency (MHRA); the drug was pure and correctly formulated [10]. It was later thought that there was up to a 4% difference in the homology of the CD28 molecule between rhesus monkeys and humans; this suggested that TGN1412 bound with a weaker affinity to the rhesus CD28. This was later disproven with reproducible results of 100% homology between the molecules.

It was not until 2010 that researchers posed a probable mechanism behind the cytokine storm in the human volunteers. As previously stated, TGN1412 selectively bound CD28 molecules on naive T cells, stimulating proliferation into T_Reg_ cells [3]. However, it was later discovered that TGN1412 also binds to CD4^+^ effector memory T cells (T_EM_) in humans [10]. CD4^+^ T_EM_ cells are memory T cells located in peripheral tissues of the body. After the body is first exposed to a pathogen, some of the previously recruited effector T cells remain at the site of the cleared infection, where they become T_EM_ cells. When activated by a second round of infection, the T_EM_ cells release pro-inflammatory cytokines, most notably TNF-α, TGF-β, and IL-2 [10].

Groundbreaking research showed that there is a distinct difference between CD4^+^ T_EM_ cells in humans and rhesus monkeys; human T_EM_ cells expressed CD28, while the rhesus T_EM_ cells did not [10]. This explains why humans had such an adverse reaction to TGN1412; the monoclonal antibody (mAb) bound to CD4^+^ T_EM_ cells to stimulate the mass release of pro-inflammatory cytokines in humans, whereas this interaction did not occur in the monkey specimen. It is important to note that these results have been reproduced only in vitro and thus may not accurately reflect the mechanisms observed in the human volunteers. However, many lines of evidence support the findings of this experiment. Most notably, the chief cytokines released in the artificial response were also largely found in the human volunteers (TNF-α, TGF-β, and IL-2) [10]. This largely supports the theory that TGN1412 binding of CD28 on human CD4^+^ T_EM_ cells caused the observed cytokine storm.

## 3. Regulation of Drug Development and Clinical Trials

The MHRA released multiple reports on the incident, and another formal report was released by an expert scientific group for improvement in future clinical trials concerning immunotherapy drugs. In the latter report (informally known as the Duff report), 22 core suggestions were provided for improvement; these were later adopted by the European Medical Agency and can be viewed in their entirety online [10,11]. This report discusses four central regulations; preclinical, clinical, and regulatory changes are discussed.

This unprecedented disaster encouraged a closer look at the protocol for carrying out safe clinical trials. While the methods current to 2006 were all adhered to for the drug development and trial, it is important that processes adapt as our knowledge of medicine advances. Drug development has undergone a notable shift towards developing immunotherapy agents; this class of drug is characterized by incredibly specific high-affinity molecular interactions. These drugs are often difficult to effectively test, particularly in animals. This is largely due to the nature of the antibody/antigen interactions, which are often not exactly reproducible in animals. For this reason, it is important that pharmacological regulations are evaluated.

The Duff report called for wider research on calculating a safe starting dose. An effective dose should be calculated using all resources available [11]. One central error of this trial was that a safe starting dose for the first clinical trial was not determined beforehand. It was later discovered that the volunteers were given close to the maximum immunostimulatory dose [12]; had they been given a safe starting dose, it is very possible that they would not have suffered such adverse reactions. A key recommendation stated that individuals must be sequentially dosed. The time between dosing of the volunteers will depend on the minimum time it would take for an adverse reaction to occur. This is called the “minimum observation period.” If an adverse reaction occurs during clinical trials, sequential dosing of volunteers could minimize the number of people affected by the drug. In the TGN1412 drug trial, the last two volunteers were injected even as the first volunteer started to experience the first signs of an adverse reaction. It would have been beneficial to wait before injecting the next volunteers.

The Duff report recommended that a treatment strategy is considered even before the volunteers are injected. This involves assessing every feasible adverse reaction that could occur after injection. A treatment would be proposed and ready, so that medical professionals could act more quickly when volunteers experience adverse reactions. A fundamental flaw in the TGN1412 drug trial was the length of time it took the volunteers to receive proper treatment. If treatment plans were constructed beforehand, the staff could have determined the cause of the reactions and could have treated them more quickly. Moreover, if treatment strategies were constructed beforehand, proper medical equipment would have been present.

The Duff report strongly asserted the need for trials to take place in an appropriate environment, conducted by adequately qualified professionals. Although this sounds an obvious regulation, the TGN1412 trial was not conducted in a hospital setting. The volunteers were not moved to an ICU until over 4 h after experiencing their first symptoms; this time is what allowed their bodily functions to deteriorate as quickly as they did. The volunteers were not diagnosed with multiple-organ failure until they reached the ICU, and it is very likely that they were experiencing this before then.

This suggests a critical improvement is needed for the way in which drug trials are conducted in Europe. A major flaw in this trial was that the volunteers received proper medical attention not when they needed it, but hours after. The volunteers needed emergency equipment immediately, but this was not available to them. This trial was conducted in a privately leased building owned by Parexel, with only basic medical equipment present. Had the volunteers been in a hospital setting with qualified medical staff, this trial might have turned out very differently. The staff might have been able to diagnose the volunteers more quickly and certainly would have been able to treat them before they reached multiple-organ failure. 

Aside from conducting the trial itself, the Duff report recommended that a standing expert advisory group be implemented for future drug trials. This team would be composed of experts in the field with different but complementary backgrounds. This team of professionals would bring diverse perspectives together in an effort to foresee disasters. For example, a pharmaceutical researcher would bring a very different perspective than an immunologist would, but together these differing views would paint a more complete picture of potential disasters. Such a team would also significantly increase communication between developers and regulators, which would be a key improvement to regulations surrounding clinical trials.

## 4. Conclusions

Key flaws of the TGN1412 drug trial were a lack of knowledge surrounding both the adverse reaction and how to treat it. The medical team were unable to pinpoint the cause of the multiple-organ failure and were therefore unsure of how to proceed with treatments. They were wholly unprepared for the disaster, which could have resulted in the death of all six volunteers if they had not been moved to the ICU when they were.

The Duff report proposed the need for additional in vitro research in drug development. The TGN1412 drug trial proceeded poorly because TGN1412 bound to T_EM_ cells as well as their target T_Reg_ cells. This is something that should have been discovered and accounted for even before in vivo studies. T_EM_ cells are known to have CD28 receptors on their surface; CD28 is a basic coreceptor molecule present on many different types of T cells. TGN1412 interaction with any cell containing CD28 should have been extensively tested to ensure that TGN1412 was only binding to the target cell.

TGN1412 re-entered clinical trials as a therapeutic for rheumatoid arthritis (renamed TAB08). A “restore” assay was developed, which more accurately assessed the response of T cells to CD28 by restoring any signal-defective T cells to their original reactivity. This assay allowed researchers to effectively analyze cytokine responses in vitro. The clinical trial was conducted by using this assay for careful analysis and by administering a significantly lower dosage to each volunteer (starting at a 1000-fold-lower dosage). Interestingly, no pro-inflammatory cytokine release was observed, and levels of IL-10, a T_Reg_ cytokine, significantly increased in each volunteer. This finding suggested good selection of T_Reg_ activation [13].

This successful clinical trial is a milestone for TGN1412, suggesting that the therapeutic is safe for use at an appropriate dosage and with proper assays for careful analysis of molecular interactions. This CD28 super antagonist has promise for development as a therapeutic for pediatric B cell leukemia, and, with these new modifications to clinical trial procedure, cytokine storm will likely be avoided in the future. Given our advancement in clinical trial protocol and newfound understanding of errors of the original trial, another look at this therapeutic could reveal a novel treatment for pediatric B cell leukemia.
